# Accurate Differentiation of Acute Aortic Syndrome from Acute Coronary Syndrome: A Machine Learning Diagnostic Model Based on Routine Admission Variables

**DOI:** 10.3390/jcm15218708

**Published:** 2026-11-07

**Authors:** Xinliang Peng, Baozhu Wang, Tiantian Tang, Suzy Wang, Xiang Ma, Christoph A. Nienaber

**Affiliations:** 1 Department of Cardiology, The First Affiliated Hospital of Xinjiang Medical University, No. 137, Liyushan South Road, Xinshi District, Urumqi 830054, China; xinliangpeng@stu.xjmu.edu.cn (X.P.); baozhuwang@xjmu.edu.cn (B.W.); 2 The First Department of Gastroenterology, The First Affiliated Hospital of Xinjiang Medical University, No. 137, Liyushan South Road, Xinshi District, Urumqi 830054, China; tiantiantang@stu.xjmu.edu.cn; 3 Hammersmith Medicines Research, Nucleus Network, 44 Cumberland Avenue, London NW10 7EW, UK; swang@hmrlondon.com; 4 Cardiology and Aortic Centre, Royal Brompton and Harefield Hospitals, Guy’s and St Thomas’ NHS Foundation Trust, London SW3 6NP, UK

**Keywords:** acute aortic syndrome, acute coronary syndrome, chest pain, machine learning, diagnostic prediction model

## Abstract

**Background/Objectives**: Acute aortic syndrome (AAS) is an underdiagnosed, time-critical medical emergency. Initial misdiagnosis as acute coronary syndrome (ACS) is common, delaying definitive treatment and exposing patients to inappropriate antithrombotic therapy. We aimed to develop and temporally test a machine learning (ML) model using routine admission variables to distinguish AAS from ACS. **Methods**: In this single-center retrospective study, we included 2828 consecutive adults with confirmed AAS or ACS (September 2018–April 2025); the initial 1979 patients formed the development cohort and the subsequent 849, a temporally separated test cohort. Least absolute shrinkage and selection operator regression selected predictors from nine prespecified candidates. Seven algorithms were fitted and assigned fixed Youden-derived cutoffs in the development cohort, then applied to the test cohort. **Results**: Six predictors were selected: D-dimer, leukocyte count, troponin I, C-reactive protein, oxygen saturation, and ST-segment changes. In the test cohort (112 AAS [13.2%]; 737 ACS), area under the receiver operating characteristic curve (AUC) ranged from 0.925 to 0.956 across the seven models. Extreme gradient boosting (XGBoost) showed the highest observed AUC (0.956; 95% confidence interval (CI), 0.935–0.976) and the lowest Brier score (0.047; 95% CI, 0.037–0.057), with sensitivity 0.884, specificity 0.931, and negative predictive value 0.981 at the development-cohort-derived cutoff (0.199); decision curve analysis indicated net benefit within the AAS/ACS test cohort. Shapley additive explanations ranked D-dimer as the dominant contributor. **Conclusions**: Six routine admission variables discriminated AAS from ACS across all seven ML algorithms in this single-center cohort. These findings support further prospective evaluation in a broader diagnostic population.

## 1. Introduction

The sudden onset of chest pain often poses a diagnostic challenge in differentiating acute aortic syndrome (AAS) from acute coronary syndrome (ACS). AAS includes acute aortic dissection (AAD), intramural hematoma (IMH), and penetrating atherosclerotic ulcer (PAU) [1]. Reported incidence rates are 10.2 in men and 5.7 in women per 100,000 person-years [2,3], compared with an estimated 7 million ACS diagnoses worldwide annually [4]. Both AAS and ACS require rapid and accurate diagnosis followed by prompt pharmacological, interventional, or surgical treatment [5,6]. Despite advances in imaging and biomarkers, AAD is still frequently misdiagnosed [7].

As ACS is more common, clinical decisions typically prioritize ACS initially [8], with the risk of inappropriate administration of antiplatelet, anticoagulant, or thrombolytic therapies, potentially harming patients with AAS [7]. ACS is among the most frequent incorrect initial diagnoses in patients with AAD [9,10,11]. In a prospective cohort of 113 patients with computed tomography angiography (CTA)-confirmed AAS, AAS was initially suspected in only 38% of cases [12]. These findings highlight the clinical challenge of distinguishing between AAS and ACS in the emergency department.

Current diagnostic strategies for AAS rely on confirmatory imaging, with electrocardiography (ECG)-gated contrast-enhanced CTA recommended as the first-line modality [13]. Transthoracic echocardiography (TTE) can assess the proximal aorta, but persistent clinical suspicion despite negative findings warrants CTA or transesophageal echocardiography (TEE) [14,15].

Among biomarkers, the International Registry of Acute Aortic Dissection (IRAD) Substudy on Biomarkers (IRAD-Bio) reported D-dimer sensitivity of 96.6% and specificity of 46.6% at a threshold of 500 ng/mL within 24 h of symptom onset [16].

The 2024 European Society of Cardiology (ESC) guidelines recommend the Aortic Dissection Detection Risk Score (ADD-RS) [1]. Because the ADD-RS relies on clinical appraisal of pain characteristics, high-risk conditions, and physical signs, its application may require substantial clinical experience. ADD-RS was evaluated in the IRAD and combined with D-dimer in the prospective ADvISED study [17,18]. In China, evidence on the use of ADD-RS combined with D-dimer to rule out AAS is limited, and early findings still require validation in larger-scale cohorts [19]. A recent meta-analysis showed that ADD-RS used alone had limited specificity, while its diagnostic performance varied with threshold definition and study population [20].

Machine learning (ML) can analyze complex nonlinear relationships among clinical and laboratory variables [21,22] and is increasingly applied in diagnosis and clinical prediction [23,24]. This study aimed to develop and temporally test an ML classification model using routine admission variables to distinguish patients with confirmed AAS from those with confirmed ACS. The present report adheres to the TRIPOD+AI reporting recommendations [25].

## 2. Materials and Methods

### 2.1. Study Design and Participants

This single-center, retrospective, observational study used data from consecutive patients admitted to the First Affiliated Hospital of Xinjiang Medical University, China, between 16 September 2018 and 18 April 2025 who presented with chest pain or related acute symptoms and received a final diagnosis of either AAS or ACS. All data were extracted from the routine electronic health-record system and included in the analysis if patients met the following criteria: (1) age ≥ 18 years and (2) chief complaints including chest pain, abdominal pain, back pain, syncope, and/or signs or symptoms suggestive of potential regional malperfusion, including myocardial, central or peripheral nervous system, mesenteric, or limb ischemia. Exclusion criteria were (1) a diagnosis of AAS or ACS confirmed at another hospital before transfer to our hospital and (2) missing candidate-predictor data despite exhaustive extraction efforts.

All patients underwent laboratory testing, electrocardiography, and vital-sign assessment (upon admission). Diagnostic confirmation was based on contrast-enhanced chest CTA for AAS and on invasive coronary angiography or coronary CTA for ACS. In patients with confirmed AAS, concomitant signs of myocardial ischemia or suspected coronary malperfusion did not change the group assignment. AAS and ACS were defined in accordance with the 2024 ESC guidelines for the management of aortic disease and the 2025 American College of Cardiology/American Heart Association (ACC/AHA) guideline for the management of ACS, respectively [1,26]. The study was conducted in accordance with the Declaration of Helsinki and was approved by the Ethics Committee of the First Affiliated Hospital of Xinjiang Medical University (approval code K202510-46; approved on 11 November 2025). The requirement for individual written informed consent was waived by the Ethics Committee due to the retrospective nature of the study.

### 2.2. Data Collection

Demographic and clinical characteristics, including age, sex, height, weight, symptoms, history of alcohol and tobacco use, comorbidities, vital signs, and ECG findings, were collected and documented upon arrival. Peripheral venous blood samples were obtained for a standard set of laboratory tests, including full blood count, D-dimer, troponin I, C-reactive protein (CRP), uric acid (UA), triglycerides (TG), and low-density lipoprotein cholesterol (LDL-C). Peripheral arterial blood was also analyzed for oxygen saturation. CTA images were interpreted by board-certified radiologists, and coronary angiograms were interpreted by senior interventional cardiologists.

Venous blood samples were processed in the hospital central laboratory. The leukocyte count was measured on a Sysmex XN-9000 automated hematology analyzer (Sysmex Corporation, Kobe, Japan). Plasma D-dimer was measured on an ACL TOP coagulation analyzer (Instrumentation Laboratory/Werfen, Bedford, MA, USA). Cardiac troponin I was measured on a VITROS 5600 Integrated System (Ortho Clinical Diagnostics, Rochester, NY, USA). CRP, UA, TG, and LDL-C were measured on a Beckman Coulter AU5800 clinical chemistry analyzer (Beckman Coulter, Brea, CA, USA). Arterial blood gas analysis, including oxygen saturation, was performed on a GEM Premier 3500 analyzer (Instrumentation Laboratory/Werfen, Bedford, MA, USA).

A 12-lead electrocardiogram was obtained in every patient on arrival and manually interpreted by clinicians blinded to the final diagnosis. ST-segment assessment was based on this single admission tracing. ST-segment changes were defined according to the Fourth Universal Definition of Myocardial Infarction [27]. ST-segment elevation was defined as new J-point elevation in two or more contiguous leads of ≥1 mm (0.1 mV) in all leads other than V2–V3. In V2–V3, the thresholds were ≥2 mm (0.2 mV) in men aged ≥40 years, ≥2.5 mm (0.25 mV) in men aged <40 years, and ≥1.5 mm (0.15 mV) in women regardless of age. ST-segment depression was defined as new horizontal or downsloping ST-segment depression of ≥0.5 mm (0.05 mV) in two or more contiguous leads. Patients meeting either criterion on the admission electrocardiogram were recorded as having ST-segment changes.

### 2.3. Data Analysis

The initial 70% of analyzed cases (from 16 September 2018 to 17 March 2024) formed the development cohort, while the subsequent 30% (from 18 March 2024 to 18 April 2025) formed the test cohort. Admission time was used only for cohort assignment and audit and was excluded from imputation, feature selection, and modeling. Missing values were imputed only for non-candidate variables, such as height and weight. A single missRanger imputation model (500 trees, maximum 10 iterations, predictive mean matching k = 5) was fitted in the development cohort with the outcome excluded. The fitted object was applied to the test cohort without refitting so that no test-cohort information contributed to imputation. Categorical variables are presented as numbers (percentages) and continuous variables as medians [interquartile ranges]. Both development and test cohort characteristics were compared using the χ^2^ or Fisher’s exact test for categorical variables and the Mann–Whitney U test for continuous variables, as appropriate. These comparisons were descriptive assessments of temporal case-mix shift and were not used to alter the test cohort or model.

Feature selection was performed exclusively in the development cohort. Nine clinically relevant prespecified candidate predictors were used: leukocyte count, D-dimer, troponin I, CRP, UA, TG, LDL-C, oxygen saturation, and ST-segment elevation or depression. After assessment of multicollinearity using the variance inflation factor (VIF), all nine variables were entered into a least absolute shrinkage and selection operator (LASSO) logistic regression model. A ten-fold stratified cross-validation was performed using the area under the receiver operating characteristic curve (AUC) as the optimization criterion, and the penalty parameter was selected with the one-standard-error rule. The variables with nonzero coefficients at λ1se were used directly for model development.

Using the selected features, seven ML models were constructed in the development cohort: logistic regression (LR), decision tree (DT), random forest (RF), extreme gradient boosting (XGBoost), Light Gradient Boosting Machine (LightGBM), support vector machine (SVM), and artificial neural network (ANN). For the six tunable machine learning algorithms, preprocessing was embedded in a pipeline, and hyperparameters were selected by grid search with stratified 5-fold cross-validation using AUC. Synthetic over-sampling, under-sampling, and class weighting were not used. After hyperparameter selection, predicted probabilities from the six machine learning models were calibrated by sigmoid (Platt) scaling using 5-fold cross-validation confined to the development cohort. LR was fitted without this additional calibration step. For each final model, the classification cutoff maximizing Youden’s J in the development cohort was fixed before evaluation and applied to the test cohort.

ML model performance was evaluated only in the test cohort. Discrimination was summarized by receiver operating characteristic (ROC) curves and AUC with 95% confidence intervals (CIs) obtained from 1000 bootstrap replicates. Overall prediction error was summarized by the Brier score with 95% bootstrap CI. Calibration was assessed graphically using locally weighted scatterplot smoothing (LOWESS) calibration curves (smoothing fraction 0.60; 1000 bootstrap replicates). Classification metrics were calculated at the fixed development-cohort cutoffs, and their 95% CIs were obtained by bootstrap without reselecting cutoffs. AUCs were compared between models by DeLong’s test for correlated ROC curves, with 95% CIs for the difference in AUC (ΔAUC) obtained by paired bootstrapping (1000 replicates). All 21 pairwise contrasts were treated as a single family and adjusted by the Holm procedure, with Benjamini–Hochberg adjustment as a sensitivity analysis; the reported ΔAUC CIs are unadjusted. These comparisons were secondary and did not inform feature selection, tuning, or cutoff derivation. Decision curve analysis (DCA) quantified net benefit over threshold probabilities of 0.01–0.50. Shapley additive explanations (SHAP) analyses were restricted to the 849 test-cohort observations and interpreted the XGBoost tree output used within the calibrated prediction workflow; SHAP was not used for feature selection, tuning, or cutoff determination.

Analyses were conducted using Python 3.10.4 (numpy 2.2.6, pandas 2.3.3, scikit-learn 1.6.1, statsmodels 0.14.6, XGBoost 3.0.2, LightGBM 4.6.0, shap 0.47.0, and matplotlib 3.10.3). Data preprocessing, missRanger single imputation, VIF calculation, and LASSO regression were performed in R 4.4.1 (missRanger 2.6.0, glmnet 4.1-8, and car 3.1-2). Two-sided *p* values < 0.05 were considered statistically significant.

## 3. Results

### 3.1. Baseline Characteristics

**Figure 1:**
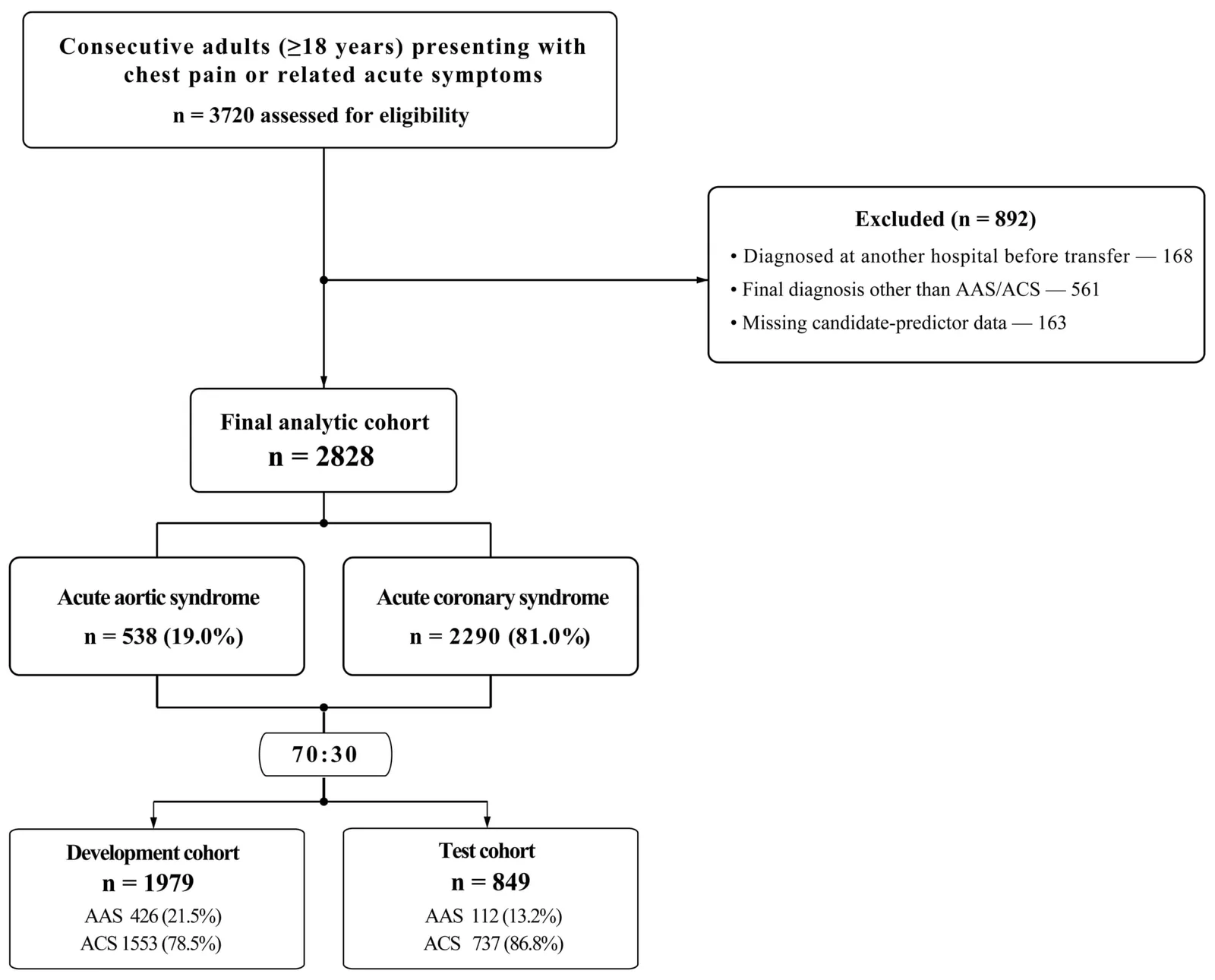
Study flow and temporal separation of the development and test cohorts. The 70:30 partition was chronological: patients were ordered by admission time; the earliest 70% (16 September 2018 to 17 March 2024) formed the development cohort, and the latest 30% (18 March 2024 to 18 April 2025) formed the temporally separated test cohort. AAS, acute aortic syndrome; ACS, acute coronary syndrome.

Of a total of 3720 patients assessed for eligibility, 892 were excluded, leaving 2828 for analysis (Figure 1). AAS was the confirmed diagnosis in 538 patients (19.0%) and ACS in 2290 (81.0%). Patients with AAS were younger than those with ACS (median [interquartile range], 55.0 [48.0–64.0] vs. 63.0 [56.0–71.0] years, respectively; *p* < 0.001), more frequently male (80.1% vs. 74.2%; *p* = 0.005), and more often had a smoking history (48.9% vs. 38.9%; *p* < 0.001). Alcohol use did not differ significantly (28.3% vs. 24.5%; *p* = 0.076). Body mass index (BMI) was modestly lower in the AAS group (median 25.39 vs. 25.68 kg/m^2^; *p* = 0.030), and ST-segment changes were less frequent (13.2% vs. 32.4%; *p* < 0.001). Patients with AAS had higher D-dimer, CRP, leukocyte count, oxygen saturation, and UA levels, whereas troponin I and triglyceride levels were higher in patients with ACS. LDL-C levels did not differ significantly between the two groups. Detailed baseline characteristics are presented in Table 1.

### 3.2. Temporal Cohort Characteristics and Feature Selection

Chronological partitioning established a development cohort of 1979 patients (16 September 2018 to 17 March 2024; with 426 AAS [21.53%] cases) and a test cohort of 849 patients (18 March 2024 to 18 April 2025; with 112 AAS [13.19%] cases). The lower AAS prevalence and differences in several clinical variables in the later test cohort reflected a moderate case-mix shift (Table S1). All nine candidate-predictor VIFs were ≤ 1.187 (Table S2). At λ1se, LASSO retained six predictors directly: ST-segment changes, leukocyte count, oxygen saturation, troponin I, CRP, and D-dimer (Figure S1).

### 3.3. Evaluation in the Test Cohort

**Figure 2:**
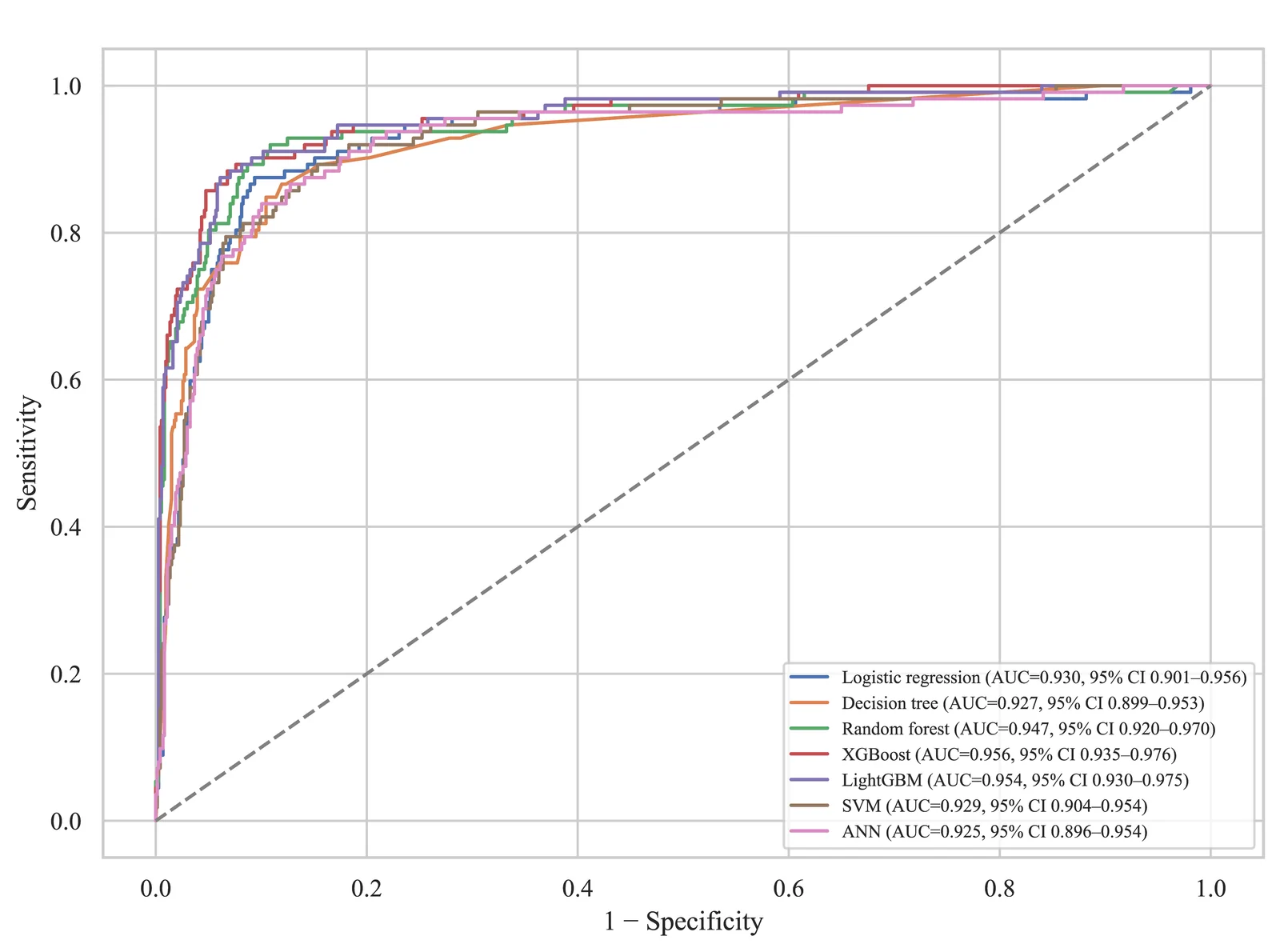
Receiver operating characteristic curves of the seven models in the temporally separated test cohort. Curves and AUCs are based exclusively on the 849 test-cohort observations. The model-specific AUC estimates and 95% bootstrap CIs are reported in Table 2. AUC, area under the receiver operating characteristic curve; CI, confidence interval.

The final models, refitted using the entire development cohort and the development-cohort-selected hyperparameters, probability-calibration procedures, and classification cutoffs, were applied without updating to the later test cohort (*n* = 849). ROC curves are shown in Figure 2, and detailed test-cohort metrics are reported in Table 2. LOWESS calibration curves for all seven models are shown in Figure S2.

Test-cohort AUCs ranged from 0.925 to 0.956. XGBoost produced the highest observed AUC (0.956; 95% CI, 0.935–0.976), followed closely by LightGBM (0.954; 95% CI, 0.930–0.975) and RF (0.947; 95% CI, 0.920–0.970), with overlapping confidence intervals. XGBoost also generated the lowest Brier score (0.047; 95% CI, 0.037–0.057), indicating the lowest overall mean squared error of the predicted probabilities among the seven models. At the development-cohort-derived cutoff of 0.199, XGBoost achieved a sensitivity of 0.884 (95% CI, 0.824–0.943), a specificity of 0.931 (0.913–0.948), a positive predictive value of 0.660 (0.582–0.736), and a negative predictive value of 0.981 (0.971–0.991); full metrics for all models are given in Table 2.

Pairwise comparisons are reported in Table S3. XGBoost discriminated better than logistic regression, the decision tree, SVM, and the ANN (ΔAUC 0.027 to 0.031; all Holm-adjusted *p* ≤ 0.020), but no statistically significant AUC difference was detected versus LightGBM (ΔAUC 0.002, 95% CI −0.001 to 0.006; *p* > 0.999) or RF (0.009, 0.001 to 0.020; *p* = 0.461); LightGBM likewise exceeded logistic regression, the decision tree, and the ANN (all Holm-adjusted *p* ≤ 0.020). Seven of the 21 contrasts were significant after adjustment, all favoring XGBoost or LightGBM, and none among the three tree-based ensembles. Clinical characteristics of all 13 false-negative AAS cases in the XGBoost test-cohort evaluation are summarized in Table S4.

**Figure 3:**
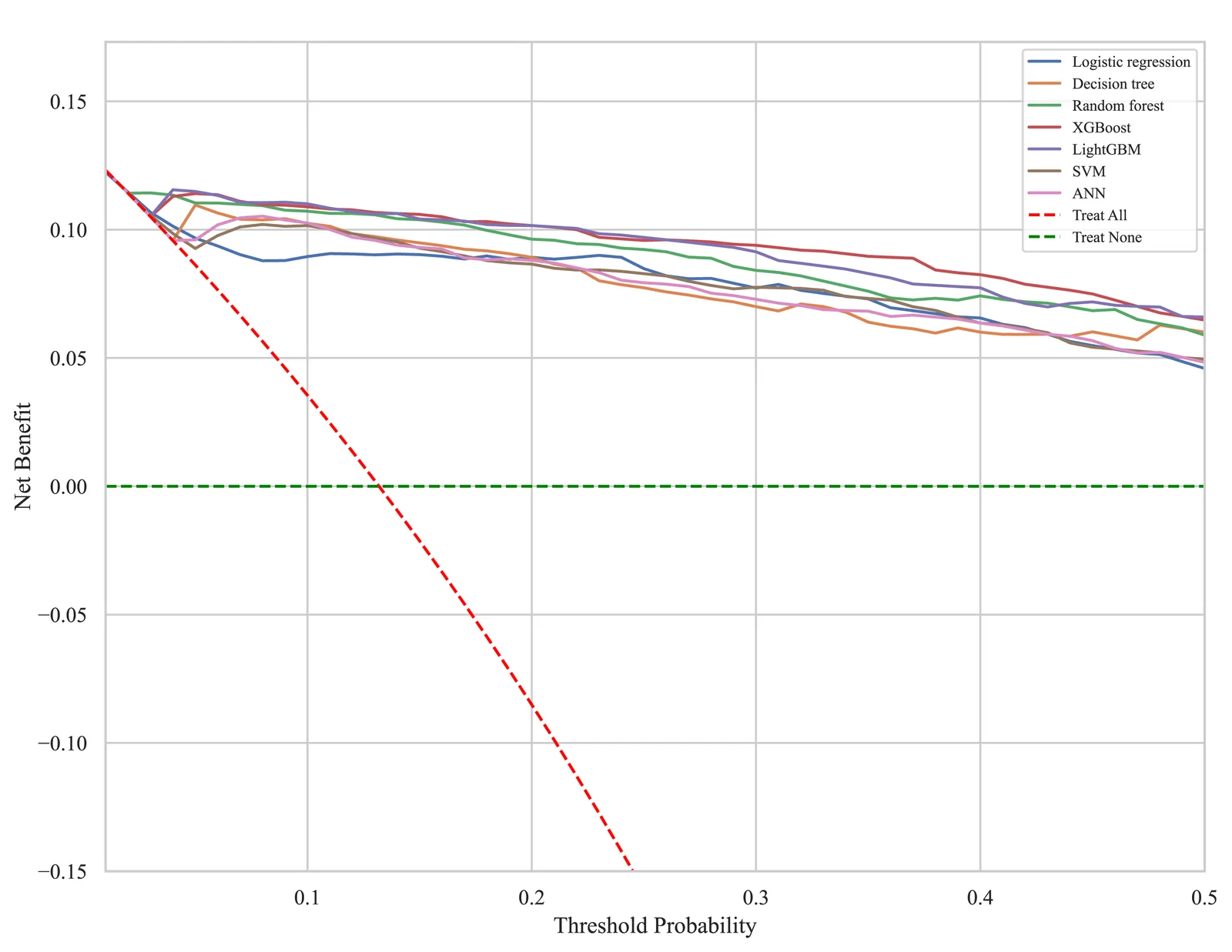
Decision curve analysis of the seven models in the test cohort (*n* = 849). Net benefit was evaluated over threshold probabilities of 0.01–0.50. The tree-based ensembles showed favorable net benefit relative to treat-all and treat-none strategies across portions of the threshold range. Curves are based exclusively on the temporally separated AAS/ACS test cohort.

In the AAS/ACS test cohort, DCA over thresholds of 0.01–0.50 showed favorable net benefit for the tree-based ensembles relative to treat-all and treat-none strategies across portions of the range (Figure 3).

### 3.4. Model Interpretability

**Figure 4:**
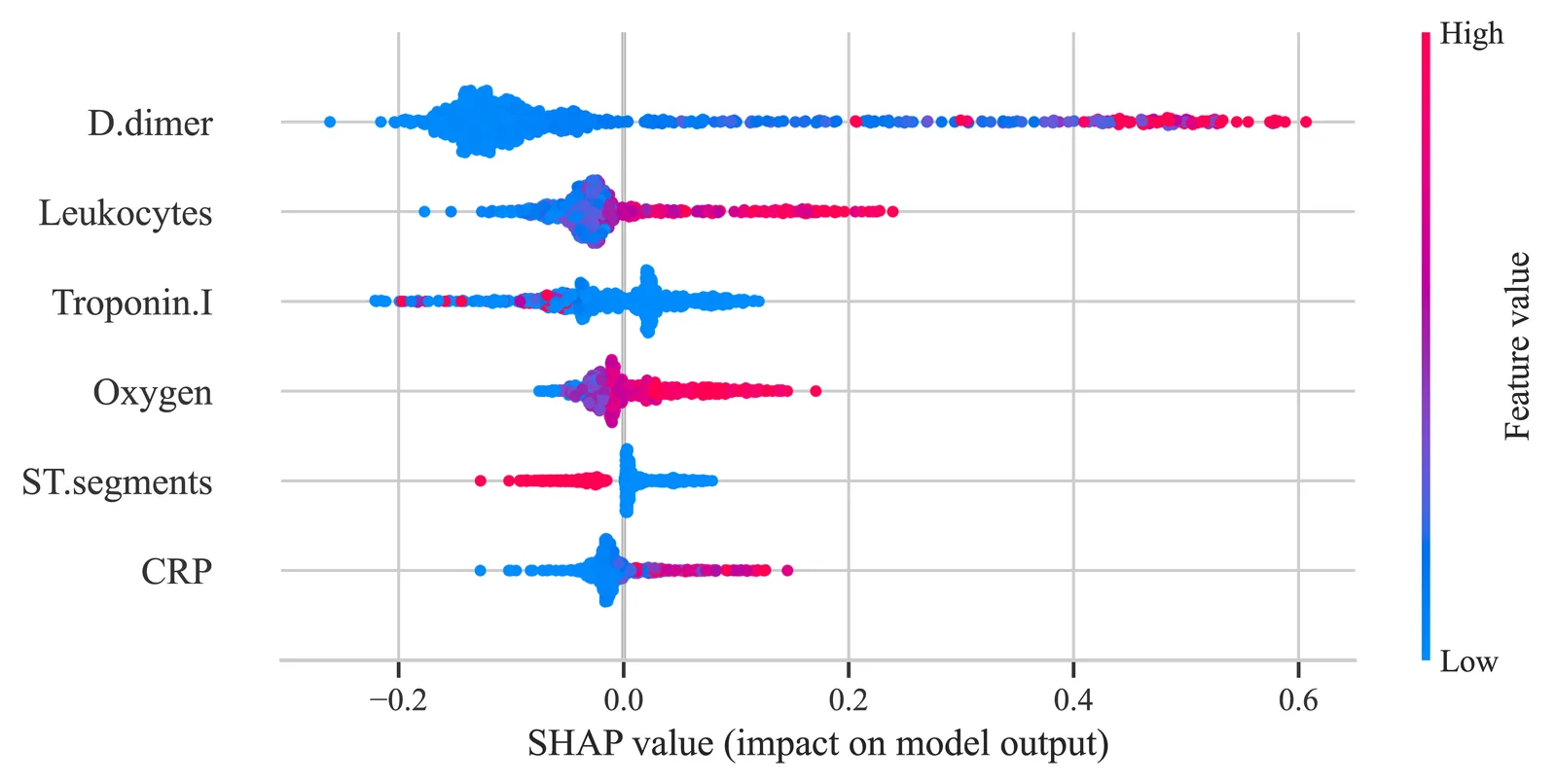
XGBoost Shapley additive explanations (SHAP) beeswarm plot based on the test cohort. Each point represents one of the 849 test-cohort observations. Red indicates higher feature values, blue indicates lower values, and the horizontal position is the SHAP contribution to the XGBoost output for AAS. D-dimer had the largest mean absolute contribution.

Global SHAP analysis of all 849 test-cohort observations ranked D-dimer as the dominant XGBoost feature (mean |SHAP| = 0.157), followed by leukocyte count (0.047), troponin I (0.046), oxygen saturation (0.032), ST-segment changes (0.022), and CRP (0.022) (Figure 4 and Figure S3). Higher D-dimer, leukocyte count, CRP, and oxygen saturation generally shifted the XGBoost output toward AAS, whereas higher troponin I and the presence of ST-segment changes shifted the output toward ACS.

**Figure 5:**
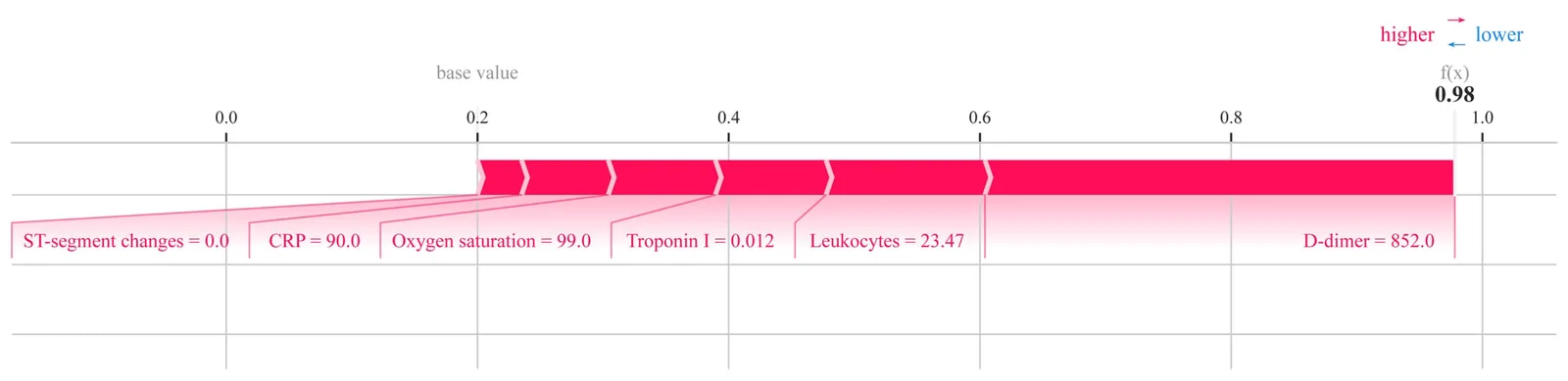
SHAP force plot for a representative patient with AAS from the test cohort (XGBoost model). The base value E[f(X)] = 0.200 is the average XGBoost tree output, and f(x) = 0.978 is the model output for this representative true AAS case. Features shown in red push the output toward AAS, and the width of each segment is proportional to that feature’s contribution. D-dimer (852 ng/mL) was the dominant contributor, with additional positive contributions from leukocyte count (23.47 × 10^9^/L), troponin I (0.012 ng/mL), oxygen saturation (99%), CRP (90.0 mg/L), and the absence of ST-segment changes.

**Figure 6:**
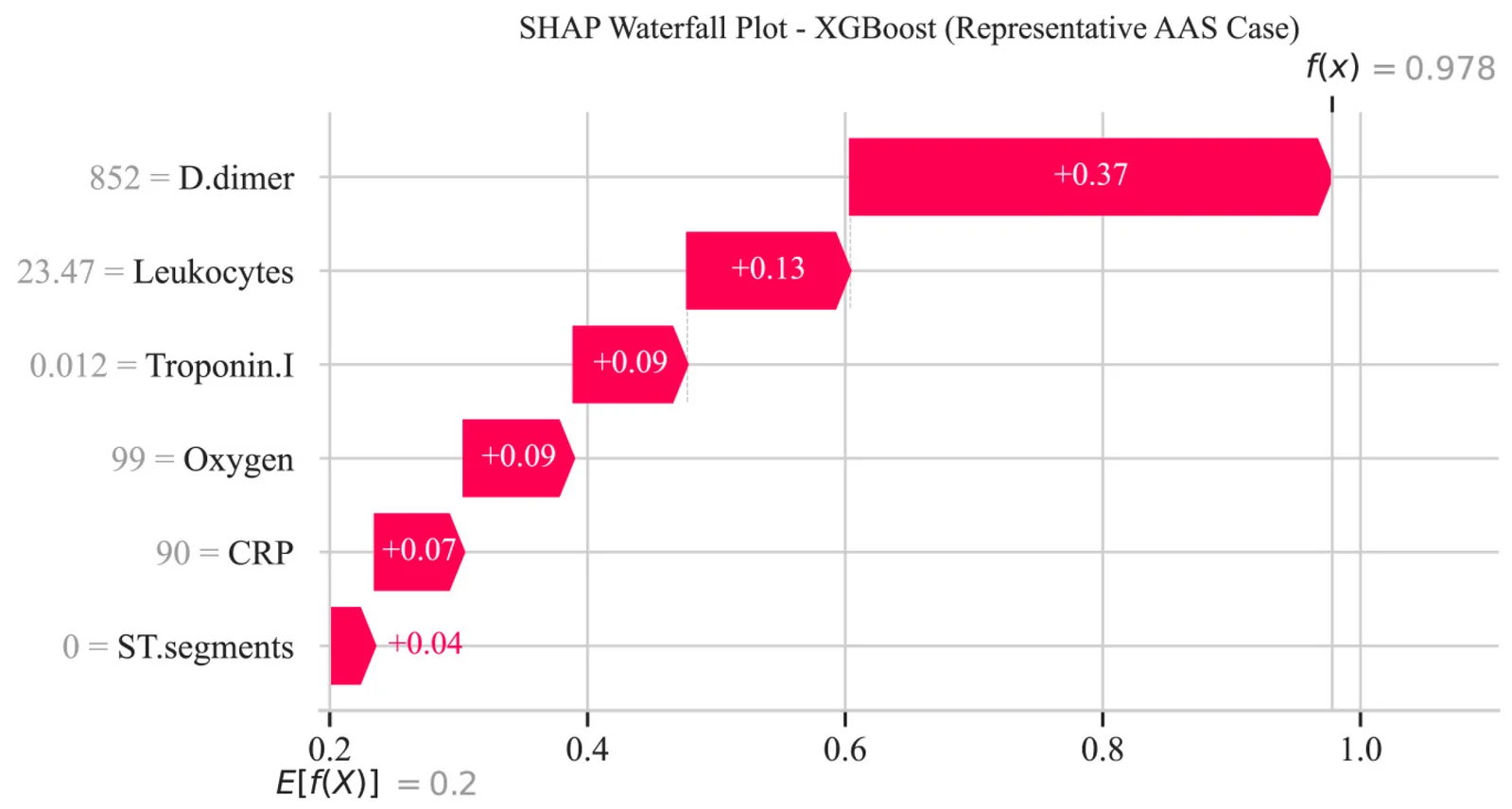
XGBoost SHAP waterfall plot for the same representative AAS case in the test cohort. The XGBoost output for this representative true AAS case increased from a base value of 0.200 to 0.978. D-dimer (+0.374) was the largest positive contribution, followed by leukocyte count (+0.126), troponin I (+0.088), oxygen saturation (+0.085), CRP (+0.069), and absence of ST-segment changes (+0.035).

Local SHAP force and waterfall plots were generated for a representative true AAS case in the test cohort whose local contribution pattern closely matched the global SHAP importance profile. The case had a D-dimer level of 852 ng/mL, leukocyte count of 23.47 × 10^9^/L, troponin I level of 0.012 ng/mL, CRP level of 90.0 mg/L, oxygen saturation of 99%, and no ST-segment changes. The XGBoost output increased from a base value of 0.200 to 0.978, driven mainly by D-dimer (+0.374), followed by leukocyte count (+0.126), troponin I (+0.088), oxygen saturation (+0.085), CRP (+0.069), and absence of ST-segment changes (+0.035) (Figure 5 and Figure 6).

## 4. Discussion

The differentiation of AAS from ACS is regularly required in the emergency department but can be challenging due to markedly different frequencies of occurrence and overlapping clinical symptoms. If AAS is misdiagnosed as ACS, potentially harmful medication or invasive procedures may be initiated, and the recognition of a life-threatening condition may be delayed. Recognition of AAS relies on clinical awareness and the interpretation of clinical signs and symptoms. The application of various algorithms using standard clinical variables obtained on admission to an emergency department showed strong discrimination between AAS and ACS, with all tested ML models reaching an AUC of 0.925–0.956 in the test cohort; the three tree-based ensembles XGBoost, LightGBM, and RF had the highest AUCs of 0.956, 0.954, and 0.947, respectively, alongside the lowest Brier scores (0.047–0.052). After adjustment for 21 pairwise comparisons, XGBoost discriminated significantly better than logistic regression, the decision tree, SVM, and the ANN, and LightGBM discriminated significantly better than logistic regression, the decision tree, and the ANN; no statistically significant AUC differences were detected among the three tree-based ensembles (Table S3). XGBoost showed the most favorable observed combination of discrimination (AUC 0.956), overall prediction error (Brier score 0.047), sensitivity (0.884), and NPV (0.981) for the under-represented AAS class and was therefore selected as the primary model for interpretation.

Our analysis has shown that all seven ML models using six routine admission variables achieved a high degree of differentiation between AAS and ACS in this cohort. These variables integrate standard admission laboratory testing with a protocol-based electrocardiographic reading. XGBoost achieved an NPV of 98.1% in the test cohort, where the AAS prevalence was 13.2%. The ongoing independent prospective multicenter PREVIEW study will further evaluate this approach in a broader diagnostic population.

From a practical standpoint, the proposed model is intended to assist differentiation between AAS and ACS once the initial laboratory results and the admission electrocardiogram are available. The development-cohort-derived threshold serves as a classification cutoff. In a future implementation, these six variables could be fed into a web-based or mobile application to provide an individualized model estimate. The global SHAP beeswarm plot and individual-level force and waterfall plots (Figure 4, Figure 5 and Figure 6) explain the relative impact of each variable on diagnostic allocation in the test cohort. Because oxygen saturation was measured by arterial blood gas analysis, implementation may be limited where arterial sampling is not routine in patients with chest pain. Pulse oximetry would be more convenient but cannot be assumed to be interchangeable without validation and will therefore be evaluated in PREVIEW.

### 4.1. Previous Studies

A series of validation studies of the ADD-RS, both in Western countries and in China, illustrates both the appeal and limitations of the score. In the original IRAD-based evaluation of the ADD-RS, the score was retrospectively applied to >2500 patients with confirmed AAD, of whom 95.7% had a score of ≥ 1 [17]. The prospective multicenter ADvISED study subsequently combined ADD-RS with D-dimer in patients with suspected AAS and identified AAS in approximately 1 in 300 patients classified as ruled out using ADD-RS = 0 or ADD-RS ≤ 1 together with D-dimer < 500 ng/mL [18]. In an Asian population, evidence and use of a clinical score are limited: a two-center Chinese cohort study found that ADD-RS combined with D-dimer yielded an AUC of 0.929, and a model using ADD-RS > 1 plus D-dimer > 2000 ng/mL achieved an NPV of 98.2% [19]; however, the authors noted that this threshold was study-specific. Delayed diagnosis of AAD remains a recognized clinical problem, with diagnostic times varying substantially across study settings and clinical presentations; factors such as atypical symptoms, ACS-like ECG findings, troponin positivity, transfer from non-tertiary hospitals, and limited clinician exposure to aortic emergencies may all contribute substantially to diagnostic delay [28]. These well-known problems motivate the investigation of routine clinical variables for differentiating AAS from ACS.

D-dimer has previously been reported as a useful biomarker, although it is not specific and may also be elevated in other thrombotic or ischemic conditions, including acute myocardial infarction, pulmonary embolism, and deep venous thrombosis [16,29,30,31]. A recent analysis has found that the neutrophil-to-lymphocyte ratio achieved diagnostic performance for AAS comparable to that of D-dimer (AUC 0.845 vs. 0.822), underscoring that even simple, routinely available markers carry discriminative information. However, any single biomarker or biomarker pair has limited discriminatory value [32].

When ML algorithms integrated six routine parameters, test-cohort AUCs ranged from 0.925 to 0.956, supporting the view that a more holistic approach including ST-segment analysis, CRP, and troponin I can support the differentiation between AAS and ACS.

### 4.2. Strengths

Our study has several strengths, including the combined use of six features obtained during routine admission assessment. Moreover, our analysis used data from a large cohort (*n* = 2828 with 538 AAS cases) with confirmed AAS or ACS over almost 7 years. Finally, patients were separated chronologically into a development cohort and a later test cohort, with seven different ML algorithms compared head-to-head in the test cohort using AUC, Brier score, LOWESS calibration curves, and decision curve net benefit as complementary metrics, with between-model differences in AUC formally tested and adjusted for multiplicity.

### 4.3. Limitations

This retrospective study has several limitations. First, this was a single-center study conducted at one large tertiary referral hospital, and both the development and test cohorts were generated within the same center; no geographic or independent external validation was performed, so generalizability to other health care systems, ethnic populations, and laboratory assay platforms has not been established. Second, the diagnostic spectrum was restricted to patients with an imaging-confirmed diagnosis of AAS or ACS; other important causes of high-risk chest pain (e.g., pulmonary embolism, musculoskeletal pain, and esophageal disease) were not modeled and will be incorporated in a forthcoming prospective analysis. DCA estimates apply to this selected population and do not establish clinical benefit in undifferentiated emergency presentations. Third, the prevalence of AAS decreased from 21.5% in the development cohort to 13.2% in the test cohort, and several predictor distributions changed over time. Our deliberate decision not to apply resampling or class weighting, although appropriate for preserving calibration, may limit sensitivity for the minority class. Fourth, requiring complete predictor data and a final diagnosis may have introduced selection and survivorship bias, including underrepresentation of patients who died before testing or diagnostic confirmation. The effect of these exclusions cannot be quantified from the available records. Fifth, details of AAS and ACS subtypes and the time of symptom onset were not consistently available in the historical records. Model performance within individual subtypes and in very early presentations therefore remains to be determined and will be assessed in the prospective PREVIEW study.

## 5. Conclusions

In conclusion, six routinely available admission variables enabled strong discrimination between patients with confirmed AAS and those with confirmed ACS by ML models, with XGBoost showing the most favorable observed combination of discriminatory power, overall prediction error, sensitivity, and negative predictive value in the same-center temporal test cohort. These findings support prospective evaluation of this approach in a broader clinical setting.

## Figures and Tables

**Table 1:** Demographic and clinical characteristics of the overall cohort.

Characteristics	Overall (*n* = 2828)	AAS (*n* = 538)	ACS (*n* = 2290)	*p*-Value
Age (years)	62.00 [54.00, 71.00]	55.00 [48.00, 64.00]	63.00 [56.00, 71.00]	<0.001
Sex, n (%)				0.005
Female	697 (24.65)	107 (19.89)	590 (25.76)	
Male	2131 (75.35)	431 (80.11)	1700 (74.24)	
Smoke, n (%)				<0.001
No	1675 (59.23)	275 (51.12)	1400 (61.14)	
Yes	1153 (40.77)	263 (48.88)	890 (38.86)	
Alcohol, n (%)				0.076
No	2116 (74.82)	386 (71.75)	1730 (75.55)	
Yes	712 (25.18)	152 (28.25)	560 (24.45)	
BMI (kg/m^2^)	25.61 [23.39, 28.28]	25.39 [22.60, 28.38]	25.68 [23.46, 28.24]	0.030
ST-segment changes, n (%)				<0.001
No	2016 (71.29)	467 (86.80)	1549 (67.64)	
Yes	812 (28.71)	71 (13.20)	741 (32.36)	
D-dimer (ng/mL)	143.00 [78.00, 375.25]	1147.50 [517.75, 3063.25]	115.00 [69.00, 210.00]	<0.001
Troponin I (ng/mL)	0.01 [0.01, 0.04]	0.01 [0.01, 0.03]	0.01 [0.01, 0.05]	0.037
CRP (mg/L)	6.60 [5.00, 15.51]	16.55 [6.23, 48.38]	5.90 [5.00, 11.40]	<0.001
Leukocytes (×10^9^/L)	7.48 [6.13, 9.50]	10.88 [8.34, 13.80]	7.10 [5.91, 8.59]	<0.001
Oxygen saturation (%)	96.00 [94.00, 98.00]	97.00 [95.00, 98.70]	96.00 [94.00, 97.00]	<0.001
UA (µmol/L)	320.49 [264.60, 388.92]	330.81 [268.90, 413.04]	318.66 [264.18, 385.05]	0.002
TG (mmol/L)	1.38 [0.98, 2.03]	1.25 [0.91, 1.84]	1.41 [1.00, 2.07]	<0.001
LDL-C (mmol/L)	2.44 [1.89, 3.09]	2.49 [2.04, 3.01]	2.42 [1.86, 3.11]	0.374

Continuous variables are presented as median [interquartile range] and compared with the Mann–Whitney U test; categorical variables are presented as numbers (percentages) and compared with the χ^2^ or Fisher’s exact test, as appropriate. AAS, acute aortic syndrome; ACS, acute coronary syndrome; BMI, body mass index; CRP, C-reactive protein; UA, uric acid; TG, triglycerides; LDL-C, low-density lipoprotein cholesterol.

**Table 2:** Performance of the models in the temporally separated test cohort (*n* = 849).

Model	AUC (95% CI)	Brier Score (95% CI)	Accuracy (95% CI)	Sensitivity (95% CI)	Specificity (95% CI)	PPV (95% CI)	NPV (95% CI)	F1 Score (95% CI)
Logistic regression	0.930 (0.901–0.956)	0.066 (0.055–0.078)	0.872 (0.849–0.894)	0.884 (0.824–0.943)	0.870 (0.847–0.893)	0.508 (0.440–0.579)	0.980 (0.969–0.991)	0.645 (0.579–0.707)
Decision tree	0.927 (0.899–0.953)	0.060 (0.050–0.071)	0.879 (0.857–0.901)	0.866 (0.802–0.928)	0.881 (0.857–0.904)	0.524 (0.449–0.602)	0.977 (0.966–0.988)	0.653 (0.586–0.715)
Random forest	0.947 (0.920–0.970)	0.052 (0.042–0.063)	0.931 (0.914–0.947)	0.723 (0.646–0.798)	0.962 (0.949–0.975)	0.743 (0.660–0.824)	0.958 (0.944–0.971)	0.733 (0.670–0.793)
XGBoost	0.956 (0.935–0.976)	0.047 (0.037–0.057)	0.925 (0.907–0.941)	0.884 (0.824–0.943)	0.931 (0.913–0.948)	0.660 (0.582–0.736)	0.981 (0.971–0.991)	0.756 (0.695–0.811)
LightGBM	0.954 (0.930–0.975)	0.049 (0.038–0.060)	0.919 (0.901–0.935)	0.884 (0.824–0.943)	0.924 (0.905–0.942)	0.639 (0.557–0.715)	0.981 (0.971–0.991)	0.742 (0.678–0.799)
SVM	0.929 (0.904–0.954)	0.066 (0.054–0.079)	0.872 (0.849–0.894)	0.848 (0.782–0.916)	0.875 (0.850–0.899)	0.508 (0.437–0.582)	0.974 (0.962–0.986)	0.635 (0.571–0.700)
ANN	0.925 (0.896–0.954)	0.066 (0.054–0.078)	0.861 (0.837–0.885)	0.866 (0.802–0.928)	0.860 (0.835–0.886)	0.485 (0.418–0.554)	0.977 (0.965–0.988)	0.622 (0.556–0.683)

All metrics were calculated exclusively in the 849 test-cohort observations, and all 95% CIs were obtained from 1000 bootstrap replicates. ANN, artificial neural network; AUC, area under the receiver operating characteristic curve; CI, confidence interval; LightGBM, Light Gradient Boosting Machine; NPV, negative predictive value; PPV, positive predictive value; SVM, support vector machine; XGBoost, extreme gradient boosting.

## Data Availability

Data containing potentially identifying patient information are not publicly available. De-identified data may be available from the corresponding author upon reasonable request.

## Supplementary Materials

The following supporting information can be downloaded at https://www.mdpi.com/article/10.3390/jcm15218708/s1, Table S1: Demographic and clinical characteristics of the development and temporally separated test cohorts; Table S2: Variance inflation factors for the nine prespecified candidate predictors in the development cohort; Table S3: Pairwise comparison of the areas under the receiver operating characteristic curve of the seven models in the test cohort; Table S4: Clinical characteristics of false-negative AAS cases in the XGBoost test-cohort evaluation; Figure S1: LASSO feature selection in the development cohort; Figure S2: LOWESS calibration curves of all seven models in the test cohort; Figure S3: Global XGBoost SHAP feature importance in the test cohort.

## Abbreviations

The following abbreviations are used in this manuscript:

AAD	acute aortic dissection
AAS	acute aortic syndrome
ACC/AHA	American College of Cardiology/American Heart Association
ACS	acute coronary syndrome
ADD-RS	Aortic Dissection Detection Risk Score
ANN	artificial neural network
AUC	area under the receiver operating characteristic curve
BMI	body mass index
CI	confidence interval
CRP	C-reactive protein
CTA	computed tomography angiography
DCA	decision curve analysis
DT	decision tree
ECG	electrocardiography
ESC	European Society of Cardiology
IMH	intramural hematoma
IRAD	International Registry of Acute Aortic Dissection
LASSO	least absolute shrinkage and selection operator
LDL-C	low-density lipoprotein cholesterol
LightGBM	Light Gradient Boosting Machine
LOWESS	locally weighted scatterplot smoothing
LR	logistic regression
ML	machine learning
NPV	negative predictive value
PAU	penetrating atherosclerotic ulcer
PPV	positive predictive value
RF	random forest
ROC	receiver operating characteristic
SHAP	Shapley additive explanations
SVM	support vector machine
TEE	transesophageal echocardiography
TG	triglycerides
TRIPOD+AI	Transparent Reporting of a multivariable prediction model for Individual Prognosis Or Diagnosis + Artificial Intelligence
TTE	transthoracic echocardiography
UA	uric acid
VIF	variance inflation factor
XGBoost	extreme gradient boosting
