# Supplementary material for: Accurate Differentiation of Acute Aortic Syndrome from Acute Coronary Syndrome: A Machine Learning Diagnostic Model Based on Routine Admission Variables
Source: J Clin Med. 2026 Nov 7;15(21):8708. doi: 10.3390/jcm15218708 (PMC13763781; doi:10.3390/jcm15218708)
Supplement: Supplementary file 1 [file jcm-15-08708-s001.zip › jcm-4605106-supplementary.pdf]

Supplementary Materials

Supplementary Materials: The following supporting information is provided with this article:

Table S1: Demographic and clinical characteristics of the development and temporally separated test cohorts.

Table S2: Variance inflation factors for the nine prespecified candidate predictors in the development cohort.

Figure S1: LASSO feature selection in the development cohort.

Figure S2: LOWESS calibration curves of all seven models in the test cohort.

Table S3: Pairwise comparison of the areas under the receiver operating characteristic curve of the seven models in the test cohort.

Table S4: Clinical characteristics of false-negative AAS cases in the XGBoost test-cohort evaluation.

Figure S3: Global XGBoost SHAP feature importance in the test cohort.

Table S1. Demographic and clinical characteristics of the development and temporally separated test cohorts.

**Characteristics**	**Overall (*n* = 2828)**	**Development cohort (*n* = 1979)**	**Temporally separated test cohort (*n* = 849)**	***p*-Value**
Diagnosis, n (%)				<0.001
AAS	538 (19.02)	426 (21.53)	112 (13.19)	
ACS	2290 (80.98)	1553 (78.47)	737 (86.81)	
Age (years)	62.00 [54.00, 71.00]	61.00 [53.00, 70.00]	63.00 [55.00, 71.00]	0.003
Sex, n (%)				0.101
Female	697 (24.65)	470 (23.75)	227 (26.74)	
Male	2131 (75.35)	1509 (76.25)	622 (73.26)	
Smoke, n (%)				0.255
No	1675 (59.23)	1158 (58.51)	517 (60.90)	
Yes	1153 (40.77)	821 (41.49)	332 (39.10)	
Alcohol, n (%)				0.210
No	2116 (74.82)	1467 (74.13)	649 (76.44)	
Yes	712 (25.18)	512 (25.87)	200 (23.56)	
BMI (kg/m^2)	25.61 [23.39, 28.28]	25.62 [23.44, 28.12]	25.56 [23.23, 28.37]	0.965
ST-segment changes, n (%)				0.098
No	2016 (71.29)	1392 (70.34)	624 (73.50)	
Yes	812 (28.71)	587 (29.66)	225 (26.50)	
D-dimer (ng/mL)	143.00 [78.00, 375.25]	154.00 [81.00, 403.00]	130.00 [77.00, 285.00]	<0.001
Troponin I (ng/mL)	0.01 [0.01, 0.04]	0.01 [0.01, 0.05]	0.01 [0.01, 0.04]	<0.001
CRP (mg/L)	6.60 [5.00, 15.51]	7.00 [5.00, 15.80]	5.50 [5.00, 14.50]	<0.001
Leukocytes (×10^9/L)	7.48 [6.13, 9.50]	7.51 [6.16, 9.59]	7.44 [6.06, 9.32]	0.206
Oxygen saturation (%)	96.00 [94.00, 98.00]	96.00 [94.00, 97.40]	96.00 [95.00, 98.00]	0.001
UA (μmol/L)	320.49 [264.60, 388.92]	325.51 [267.02, 397.37]	311.65 [259.22, 375.39]	<0.001
TG (mmol/L)	1.38 [0.98, 2.03]	1.36 [0.96, 2.04]	1.42 [1.03, 1.98]	0.068
LDL-C (mmol/L)	2.44 [1.89, 3.09]	2.42 [1.86, 3.05]	2.52 [1.96, 3.19]	0.002

Patients were ordered by admission time. The earliest 1979 patients formed the development cohort and the latest 849 formed the temporally separated test cohort. The lower AAS prevalence and differences in several variables in the later cohort are reported as temporal case-mix shift rather than as evidence of cohort equivalence. AAS, acute aortic syndrome; ACS, acute coronary syndrome; BMI, body mass index; CRP, C-reactive protein; UA, uric acid; TG, triglycerides; LDL-C, low-density lipoprotein cholesterol.

Table S2. Variance inflation factors for the nine prespecified candidate predictors in the development cohort.

**Variable**	**VIF**
ST-segment changes	1.019
D-dimer	1.075
Troponin I	1.043
CRP	1.128
Leukocytes	1.187
Oxygen saturation	1.003
UA	1.046
TG	1.048
LDL-C	1.027

All VIF values were ≤ 1.187, indicating no problematic multicollinearity; all nine candidates were entered into LASSO regression. VIF, variance inflation factor; LASSO, least absolute shrinkage and selection operator.

(A)

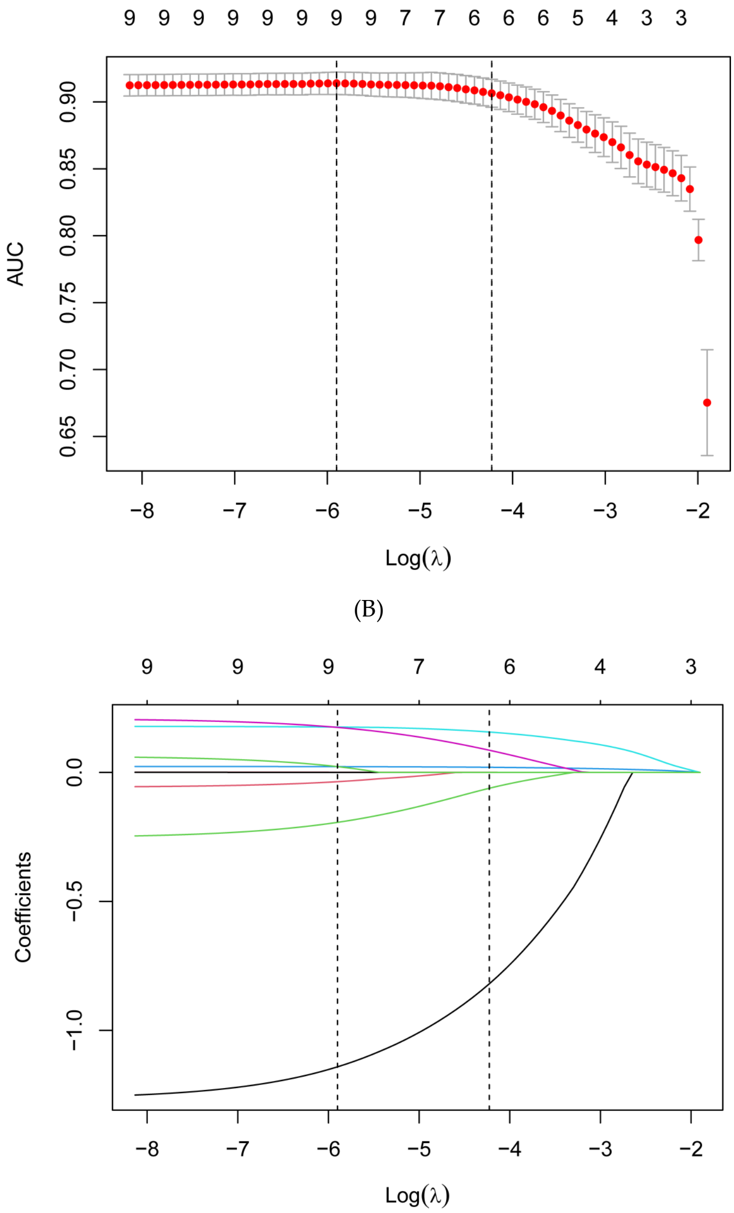

Figure S1. LASSO feature selection in the development cohort. (A) Ten-fold stratified cross-validated AUC profile of LASSO logistic regression fitted using the nine prespecified candidates; dashed lines indicate λmin and λ1se. (B) Coefficient paths across log(λ). Six predictors had nonzero coefficients at λ1se and were used directly for model development: ST-segment changes, leukocyte count, oxygen saturation, troponin I, CRP, and D-dimer. LASSO, least absolute shrinkage and selection operator; AUC, area under the receiver operating characteristic curve.

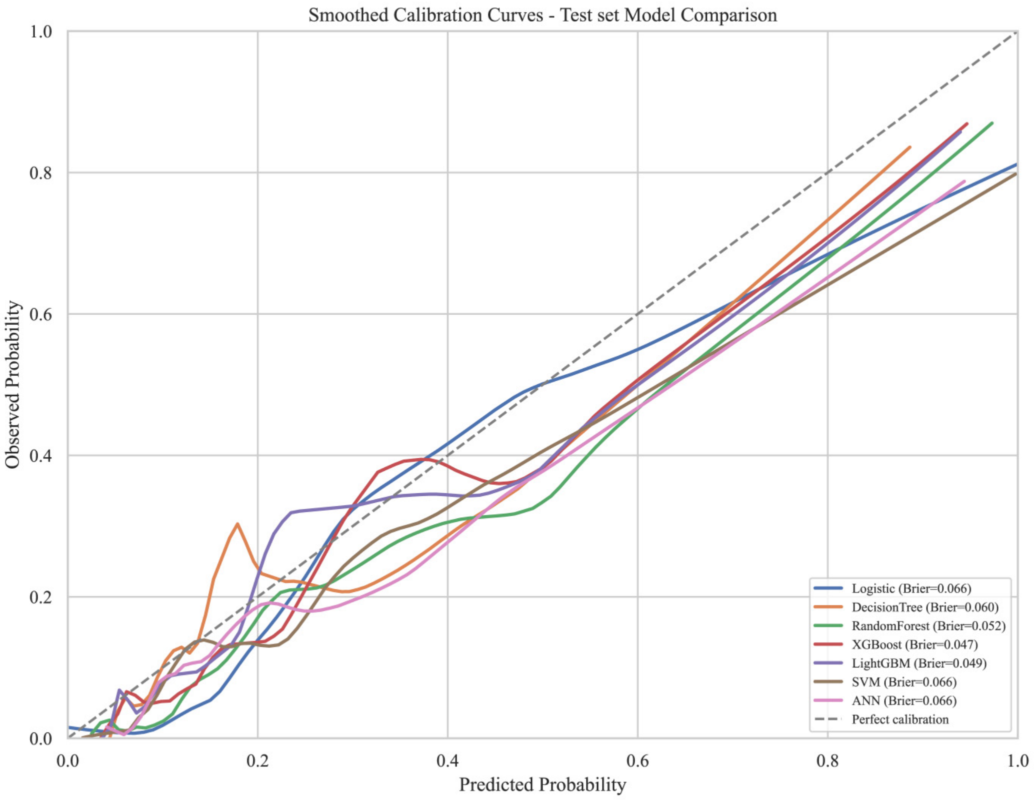

Figure S2. LOWESS calibration curves of all seven models in the test cohort. Curves compare predicted and observed AAS probabilities in the 849 test-cohort observations. Each colored curve is LOWESS-smoothed (fraction 0.60), and the diagonal denotes perfect calibration. Brier scores are shown in the legend. LOWESS, locally weighted scatterplot smoothing.

Table S3. Pairwise comparison of the areas under the receiver operating characteristic curve of the seven models in the test cohort.

**Comparison**	**AUC, Model 1**	**AUC, Model 2**	**ΔAUC**	**Bootstrap 95% CI**	**Bootstrap *p***	**DeLong *Z***	**DeLong *p***	**Holm *p***	**BH-FDR *p***
LR vs. DT	0.930	0.927	+0.003	−0.019 to +0.026	0.858	0.213	0.831	>0.999	0.921
LR vs. RF	0.930	0.947	−0.017	−0.031 to −0.003	0.020	−2.272	0.023	0.254	0.040
LR vs. XGBoost	0.930	0.956	−0.027	−0.044 to −0.012	<0.002	−3.289	0.001	0.018	0.004
LR vs. LightGBM	0.930	0.954	−0.025	−0.040 to −0.011	<0.002	−3.231	0.001	0.020	0.004
LR vs. SVM	0.930	0.929	+0.001	−0.019 to +0.016	0.874	0.074	0.941	>0.999	0.941
LR vs. ANN	0.930	0.925	+0.004	−0.001 to +0.010	0.122	1.491	0.136	>0.999	0.204
DT vs. RF	0.927	0.947	−0.020	−0.037 to −0.004	0.016	−2.272	0.023	0.254	0.040
DT vs. XGBoost	0.927	0.956	−0.029	−0.042 to −0.017	<0.002	−4.350	<0.001	<0.001	<0.001
DT vs. LightGBM	0.927	0.954	−0.027	−0.041 to −0.014	<0.002	−3.833	<0.001	0.003	0.001
DT vs. SVM	0.927	0.929	−0.002	−0.022 to +0.018	0.902	−0.174	0.862	>0.999	0.921
DT vs. ANN	0.927	0.925	+0.002	−0.022 to +0.026	0.836	0.154	0.878	>0.999	0.921
RF vs. XGBoost	0.947	0.956	−0.009	−0.020 to −0.001	0.024	−1.949	0.051	0.461	0.083
RF vs. LightGBM	0.947	0.954	−0.007	−0.018 to +0.002	0.134	−1.426	0.154	>0.999	0.215
RF vs. SVM	0.947	0.929	+0.018	+0.004 to +0.031	0.010	2.496	0.013	0.151	0.026
RF vs. ANN	0.947	0.925	+0.022	+0.007 to +0.036	0.004	2.744	0.006	0.085	0.016
XGBoost vs. LightGBM	0.956	0.954	+0.002	−0.001 to +0.006	0.250	1.104	0.270	>0.999	0.354
XGBoost vs. SVM	0.956	0.929	+0.027	+0.012 to +0.043	0.002	3.220	0.001	0.020	0.004
XGBoost vs. ANN	0.956	0.925	+0.031	+0.015 to +0.050	<0.002	3.452	<0.001	0.011	0.004
LightGBM vs. SVM	0.954	0.929	+0.025	+0.008 to +0.042	0.006	2.697	0.007	0.091	0.016
LightGBM vs. ANN	0.954	0.925	+0.029	+0.014 to +0.047	<0.002	3.296	<0.001	0.018	0.004
SVM vs. ANN	0.929	0.925	+0.004	−0.011 to +0.022	0.668	0.438	0.661	>0.999	0.817

ΔAUC = AUC(Model 1) − AUC(Model 2); positive values favor Model 1. Paired bootstrap analyses used 1,000 replicates; zero empirical tail counts are reported as $p < 0.002$ (minimum nonzero $p = 0.002$). Bootstrap 95% CIs are unadjusted and descriptive. DeLong p values for the 21 pairwise comparisons were Holm-adjusted, with Benjamini–Hochberg false discovery rate adjustment as a sensitivity analysis; main-text significance is based on Holm-adjusted p values. Comparisons were secondary. DeLong and paired bootstrap tests agreed on unadjusted significance in 20 of 21 comparisons, except XGBoost vs. RF. ANN, artificial neural network; AUC, area under the receiver operating characteristic curve; BH-FDR, Benjamini–Hochberg false-discovery rate; CI, confidence interval; DT, decision tree; LightGBM, Light Gradient Boosting Machine; LR, logistic regression; RF, random forest; SVM, support vector machine; XGBoost, extreme gradient boosting.

Table S4. Clinical characteristics of false-negative AAS cases in the XGBoost test-cohort evaluation.

Case	Age (years)	Sex	D-dimer (ng/mL)	Troponin I (ng/mL)	CRP (mg/L)	Leukocytes (×10⁹/L)	Arterial oxygen saturation (%)	ST-segment changes	Predicted AAS probability
FN01	50	F	227	<0.012	<5.00	6.45	95	Yes	0.0415
FN02	82	F	365	<0.012	<5.00	4.07	96	No	0.0589
FN03	51	M	83	<0.010	21.4	6.62	97	No	0.0483
FN04	66	M	641	0.044	85.3	8.58	95	Yes	0.1258
FN05	37	M	82	<0.012	7.6	18.40	97	No	0.0634
FN06	73	M	517	0.109	14.6	5.82	99	No	0.1673
FN07	47	M	96	<0.012	7.1	9.08	95	No	0.0391
FN08	46	M	311	0.029	84.1	9.58	97	Yes	0.0627
FN09	53	M	60	<0.012	<5.00	6.95	77	No	0.0363
FN10	53	M	53	<0.012	<5.00	7.26	94	No	0.0368
FN11	72	M	122	<0.010	<5.00	7.36	99	No	0.0761
FN12	61	M	87	<0.010	18.0	9.24	94	No	0.0399
FN13	46	M	80	<0.010	<5.00	7.76	99	No	0.0696

False negatives were AAS cases classified as ACS at the fixed XGBoost cutoff (0.199, rounded). AAS, acute aortic syndrome; ACS, acute coronary syndrome; CRP, C-reactive protein; F, female; M, male.

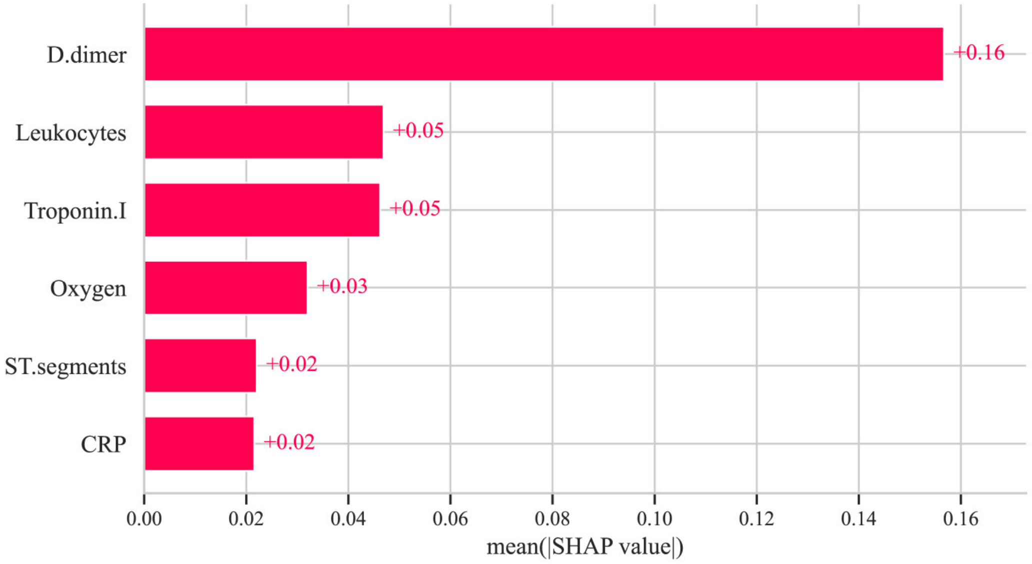

Figure S3. Global XGBoost SHAP feature importance in the test cohort. Mean absolute SHAP values across all 849 test-cohort observations were 0.157 for D-dimer, 0.047 for leukocyte count, 0.046 for troponin I, 0.032 for oxygen saturation, 0.022 for ST-segment changes, and 0.022 for CRP. SHAP values explain the XGBoost tree output and do not imply causal effects. SHAP, Shapley additive explanations.
